# Symbiont type and environmental factors affect transcriptome‐wide gene expression in the coral *Montipora capitata*

**DOI:** 10.1002/ece3.4756

**Published:** 2018-12-27

**Authors:** Martin Helmkampf, M. Renee Bellinger, Monika Frazier, Misaki Takabayashi

**Affiliations:** ^1^ Tropical Conservation Biology and Environmental Science University of Hawaiʻi at Hilo Hilo Hawaii; ^2^ Okinawa Institute of Science and Technology Onna-son, Okinawa Japan

**Keywords:** corals, gene expression, RNA‐seq, *Symbiodinium*, symbiosis, transcriptome

## Abstract

Reef‐building corals may harbor genetically distinct lineages of endosymbiotic dinoflagellates in the genus *Symbiodinium*, which have been shown to affect important colony properties, including growth rates and resilience against environmental stress. However, the molecular processes underlying these differences are not well understood. In this study, we used whole transcriptome sequencing (RNA‐seq) to assess gene expression differences between 27 samples of the coral *Montipora capitata *predominantly hosting two different *Symbiodinium *types in clades C and D. The samples were further characterized by their origin from two field sites on Hawai‘i Island with contrasting environmental conditions. We found that transcriptome‐wide gene expression profiles clearly separated by field site first, and symbiont clade second. With 273 differentially expressed genes (DEGs, 1.3% of all host transcripts), symbiont clade had a measurable effect on host gene expression, but the effect of field site proved almost an order of magnitude higher (1,957 DEGs, 9.6%). According to SNP analysis, we found moderate evidence for host genetic differentiation between field sites (*F*_ST_ = 0.046) and among corals harboring alternative symbiont clades (*F*_ST_ = 0.036), suggesting that site‐related gene expression differences are likely due to a combination of local adaptation and acclimatization to environmental factors. The correlation between host gene expression and symbiont clade may be due to several factors, including host genotype or microhabitat selecting for alternative clades, host physiology responding to different symbionts, or direct modulation of host gene expression by *Symbiodinium*. However, the magnitude of these effects at the level of transcription was unexpectedly small considering the contribution of symbiont type to holobiont phenotype.

## 1 INTRODUCTION

Symbiosis, the persistent, intimate association between species (Bouchard, 2009), shapes all aspects of the biosphere from molecular structure to ecosystem function. Most, if not all, animals engage in complex interactions with microbial symbionts and depend on them for nutrition, defense, and development (Chaston & Goodrich‐Blair, 2010). Indeed, the emerging view of animals as holobionts—ecosystems consisting of a multicellular host and associated microbial communities—has sparked new insights into genome evolution, development, metabolism, organismal health, and ecology (McFall‐Ngai et al., 2013).

Scleractinian corals are one of the most prominent examples of animal–microbe symbiosis and hold particular ecological, economic, and cultural importance (Cesar & van Beukering, 2004). In the coral holobiont, dinoflagellate algae of the genus *Symbiodinium *provide organic nutrients, oxygen, and energy derived from photosynthetic activity to the coral host in return for inorganic nutrients, carbon dioxide, and protection (Stat, Carter, & Hoegh‐Guldberg, 2006). A host of genetically and ecologically distinct species (“types”) of *Symbiodinium *belonging to several divergent lineages (“clades”) have been identified as endosymbionts in scleractinian corals (Baker, 2003; LaJeunesse et al., 2018; Weber & Medina, 2012). While in many coral species colonies associate with a single dominant type of *Symbiodinium*, others are able to host multiple types simultaneously, or in temporal succession (Baker, 2003; Berkelmans & van Oppen, 2006). The composition of the *Symbiodinium *community may influence important host and holobiont properties, including colony growth rates (Jones & Berkelmans, 2010; Mieog et al., 2009), nutrient transfer (Loram, Trapido‐Rosenthal, & Douglas, 2007), local adaptation (e.g., with regard to light exposure and depth, Iglesias‐Prieto, Beltrán, LaJeunesse, Reyes‐Bonilla, & Thomé, 2004), fitness, and tolerance of environmental stress (Berkelmans & van Oppen, 2006; Iglesias‐Prieto et al., 2004; Mieog et al., 2009). How *Symbiodinium *clades impact holobiont resilience has received particular attention. Some types, most notably in clade D, may provide the holobiont with increased tolerance of elevated temperatures and can reduce susceptibility to coral bleaching (Berkelmans & van Oppen, 2006; Stat & Gates, 2011), the breakdown of the coral–*Symbiodinium *symbiosis which is emblematic of the current coral health crisis.

Genome‐wide analyses of host gene expression hold great promise to reveal the genetic underpinnings of these functional differences. Host transcriptional changes have been studied in nonpathogenic symbioses of bacteria and vertebrates (Rawls, Samuel, & Gordon, 2004), insects (Wilson et al., 2006), cephalopods (Chun et al., 2008; Wier et al., 2010), deep‐sea mussels (Boutet et al., 2011), and polychaetes (Nyholm, Robidart, & Girguis, 2008). In cnidarians, most transcriptomic studies have focused on the establishment of symbiosis in coral larvae (Mohamed et al., 2016; Schnitzler & Weis, 2010; Voolstra et al., 2009), recently settled coral polyps (O'Rourke et al., 2017), and adult symbiotic and nonsymbiotic sea anemones (Lehnert et al., 2014; Rodriguez‐Lanetty, Phillips, & Weis, 2006). In most of these studies, significant changes in host gene expression suggested an active role of the host in the establishment and regulation of symbiosis. However, only few studies have investigated the transcriptional response of cnidarian hosts to different symbiont types. The first to show a correlation between *Symbiodinium *type and host transcription, DeSalvo et al. (2010) reported expression changes in genes involved in protein metabolism in the coral *Montastraea faveolata*. This observation was more recently corroborated in *Acropora millepora* by Barfield, Aglyamova, Bay, & Matz (2018), who demonstrated that both the symbiont type and the native reef environment result in host gene expression differences in a common garden experiment. Modulation of host physiology, including metabolism, stress response, and immune system, was also found in the sea anemone *Exaiptasia pallida* during colonization by an opportunistic *Symbiodinium *type, with respect to the dominant type (Matthews et al., 2017).

The objective of the present study was to expand upon this incipient knowledge of how different coral–*Symbiodinium *partnerships affect gene expression, and to shed more light on how interactions between symbiont partners are regulated at the genetic level in *Montipora capitata*, one of the most common coral species in Hawai‘i. Using whole transcriptome sequencing (RNA‐seq), we examined the transcriptional profiles of *M. capitata *colonies in field populations harboring *Symbiodinium *types representing clades C and D. We compared these profiles to the transcriptomic states corresponding to two other variables, field site and disease status. The two field sites, while both located on Hawai‘i Island, differed markedly with respect to underwater topography and climatic conditions, especially rainfall and temperature. Organisms can respond to environmental changes through local adaptation (allele frequency changes between generations due to natural selection) and acclimatization (nongenetic, usually short‐term phenotypic responses), both of which can manifest in gene expression changes. Environmentally sensitive genes may comprise a significant portion of the transcriptome (5%–15%; Santo et al., 2013; Zhou, Campbell, Stone, Mackay, & Anholt, 2012). The third variable we included was the presence or absence of Growth Anomaly (GA), a widespread coral disease characterized by abnormally enlarged skeletal growth. In a previous study partially based on the same dataset, we found that GA has a surprisingly limited impact on gene expression (Frazier, Helmkampf, Bellinger, Geib, & Takabayashi, 2017). Comparing the effects of field site and disease status to clade‐related gene expression differences in the present study allowed us to evaluate the magnitude of these effects. Further, we measured genetic differentiation between field sites and among corals harboring alternative clades directly from the RNA‐seq data to assess the role of host genotype. We also provided a list of host genes whose expression was correlated with *Symbiodinium *clade, and employed coexpression network analysis to illuminate the connectivity and function of genes that might be responsive to the presence of different symbiont clades.

## 2 MATERIALS AND METHODS

### 2.1 Description of field sites, sample collection, and sequencing

Sample collection, RNA extraction, and RNA sequencing were conducted as described previously (Frazier et al., 2017). Briefly, 27 core samples (*c.* 1 cm^3^) of *M. capitata *were collected in January and February 2013 from two field sites on Hawai‘i Island: Waiʻōpae (19°29′55″ N, 154°49′06″ W) and Kīholo (19°51′9″ N, 155°55′55″ W). Although only 125 km apart, and thus sharing similar latitudinal and longitudinal positions, the environmental conditions at these two sites were very different. The leeward Western coast of Hawaiʻi Island, where Kīholo Bay is situated, is arid, receiving <25 cm rainfall annually. In comparison, Waiʻōpae was located on the windward Eastern coast of Hawai‘i Island, which receives more than 200 cm precipitation per year (Annual Precipitation Averages and Extremes: Hawaii, Western Regional Climate Center). The shallow water environment of Kīholo is typical of an incipient fringing reef with gentle reef slopes covering an open bay down to approximately 10 m in depth (Johnson & Wiegner, 2013). Waiʻōpae, while on an open coastline, was composed of numerous permanently and temporarily submerged basaltic tidepools, ranging in size and connectivity at low tide (*c.* 1 m^3^ to 2,500 m^3^, 0.25–3 m depth). Such complex underwater topography at Waiʻōpae, along with high rainfall and groundwater discharge, created high variability in temperature and salinity (Wiegner, Mokiao‐Lee, & Johnson, 2016).

The 27 core samples originated from a total of 18 colonies (12 from Waiʻōpae and six from Kīholo) found at a depth between 0.5 and 3.0 m relative to mean lower low water. Half of the colonies at each site were morphologically healthy, in which case one sample was taken per colony. The other half was visibly affected by the coral disease GA. From each diseased colony, two samples were taken, one each from lesioned and morphologically unaffected tissue. In the laboratory, coral tissue was scraped off the cores using a sterile razor, and total RNA was extracted from pulverized tissue using a combination of TRIzol (Invitrogen), chloroform, and the RNeasy Mini Kit (Qiagen) following the manufacturer's instructions. Total RNA was sent to the Yale Center for Genomic Analysis for mRNA isolation and sequencing on three lanes of an Illumina HiSeq 2000. Libraries were constructed and multiplexed as described in Frazier et al. (2017), and paired‐end sequencing was conducted for 75 cycles for each read pair.

### 2.2 Transcriptome assembly and separation

The assembly process has also been described in more detail elsewhere (Frazier et al., 2017). In short, 25 million paired‐end reads were generated on average per sample (range: 17–42 million). A meta‐transcriptome representing the host and associated microorganisms was assembled from the quality‐filtered raw reads using the Trinity package (Grabherr et al., 2011). Of initially 660,340 isotigs (transcripts), 87,085 were retained after removing low expression transcripts (FPKM <0.5 summed across all samples, see below) and transcripts without open reading frames. To separate coral host from symbiont transcripts, this dataset was subjected to three processes: (a) ortholog detection using InParanoid version 4.1 (Sonnhammer & Ostlund, 2014), with genomes and transcriptomes of the coral *Acropora digitifera *(Shinzato et al., 2011) and clade B symbiont *Symbiodinium minutum* (Shinzato et al., 2011; Shoguchi et al., 2013) serving as references; (b) ortholog detection based on reciprocal best hits to cnidarian sequences in GenBank using BLASTp; and (c) retention of transcripts with a GC content ≤47% after a strong GC content bias was observed in independently annotated coral versus symbiont sequences. Transcripts that were classified as coral or cnidarian by the first two homology methods, and unclassified transcripts meeting the GC content cutoff, were regarded as *M. capitata *transcripts, resulting in a coral host transcriptome reference of 20,461 sequences.

### 2.3 *Symbiodinium* clade determination

To determine the predominant *Symbiodinium* clade in each sample, we searched the meta‐transcriptome assembly (all isotigs before quality filtering) for transcripts of the internal transcribed spacer 2 (ITS2) rDNA region using BLASTn. As queries, we selected previously described ITS2 sequences representing clades A–D (LaJeunesse, 2001: AF333505, AF333511; Putnam, Stat, Pochon, & Gates, 2012: HE578979, HE579036). Four meta‐transcriptome transcripts were found at an e‐value <0.001, one having high similarity to clade C, and three to clade D. Two of the latter differed only in flanking sequence and were identical regarding ITS2, and one was discarded because it appeared to be misassembled (the sequence homologous to ITS2 was not flanked by 28S, in addition to being lowly expressed). The two remaining sequences, referred to as reference transcripts from here on, were identified to type level by aligning them separately to ITS2 sequences of a wide diversity of types within each clade (C1, C3, C15, C17, C21, C31 for clade C; D1a, D8, D12, D13, D15 for clade D; most were taken from Putnam et al., 2012; Stat et al., 2011) with MAFFT version 7 (Katoh & Standley, 2013). Reference transcripts were then identified by their closest phylogenetic affiliation using RAxML version 8.1.20 (Stamatakis, 2014) with the GTRCAT model and 250 rapid bootstrap replicates. The composition of the *Symbiodinium *assemblage in each sample was estimated by mapping quality‐filtered raw reads to the two reference transcripts using bowtie version 2.2.4 (Langmead & Salzberg, 2012) and the ‐‐very‐sensitive‐local option. The number of uniquely mapped reads was counted for each reference transcript (read pairs were counted only once, regardless of whether both mates aligned concordantly or discordantly, or whether only one mate aligned), and per‐sample clade composition expressed as the proportion of reads mapping to the clade C or D reference. Reads that did not perfectly match the reference transcripts, suggesting the presence of intragenomic variants, alternative *Symbiodinium *types or sequencing errors, were identified by the “XM” flag in SAM output files, and their genetic distance estimated in terms of nucleotide differences.

### 2.4 Gene expression analyses

The abundance of coral host transcripts was measured in counts and FPKM values by the RSEM method (Li & Dewey, 2011) in Trinity. Subsequent analyses were performed in R version 3.2.2 (scripts are available as Supporting Information Appendix S1), using different sample sets: (a) all samples (“all”); (b) all samples except outliers (“default,” see below); (c) samples from Wai‘ōpae only (“Wai‘ōpae,” also excluding outliers); and (d) samples from healthy and morphologically GA‐unaffected tissue only (“HU,” also excluding outliers). In all cases, transcripts with a minimum count of 1 in fewer than three samples were first removed (note this filtering step was conducted in addition to the removal of lowly expressed genes during meta‐transcriptome assembly processing as described above), and counts normalized to counts per million (CPM) using edgeR version 3.10.5 (Robinson & Oshlack, 2010). Outliers were identified by hierarchical clustering of CPM distances between all samples, leading to the exclusion of sample WA8. In addition, we also defined two samples with relative ITS2 read abundances of ≥0.25 for more than one *Symbiodinium *clade as outliers (WH8 and WA12), to allow unambiguous binary coding of clade in the following analyses. Gene expression profiles were visualized by metric multidimensional scaling (MDS) of logCPM values using limma's plotMDS function (Ritchie et al., 2015) with default settings.

For differential gene expression (DGE) analyses of the default and Wai‘ōpae datasets, we first applied TMM normalization to read counts to account for composition bias, and tested for differentially expressed genes (DEGs) using limma. Three binary comparisons were set up separately, where applicable: sampling site (Waiʻōpae vs. Kīholo), dominant *Symbiodinium *clade (C vs. D), and GA disease status (healthy vs. diseased colony, where lesioned and morphologically unaffected tissues were combined). Data were voom‐transformed, a linear model fit to each gene, and model statistics estimated by the eBayes function. Differences with a FDR‐adjusted *p*‐value <0.05 were considered significant. Mean‐difference plots to illustrate DEGs were created with limma's plotMD function. Overlap in DEGs between comparisons was calculated using the Venn diagram tool available at http://bioinformatics.psb.ugent.be/webtools/Venn/.

Gene coexpression networks were examined using WGCNA version 1.51 (Langfelder & Horvath, 2008), using the default and Waiʻōpae datasets. After filtering out transcripts with a minimum FPKM of 1 in fewer than three samples, adjacency matrices were calculated based on log‐transformed FPKM values using an exponent of 14 (default dataset) and 18 (Waiʻōpae dataset), respectively. These exponents represented the lowest values for which the scale‐free topology fit index curves flattened out around 0.8. Network construction and module detection were carried out by hierarchical clustering with default settings. In addition, connectivity and module membership were calculated for each gene. The relationship between modules and external factors (site, clade, and disease status) was investigated by linear regression, with the module eigengenes serving as dependent variables. FDR‐adjusted *p*‐values were calculated to account for multiple testing.

### 2.5 Gene annotation

Translated coral host genes were annotated using (a) HMMER v3.1b2 (hmmer.org) searches against the Pfam‐A database v29 (Finn et al., 2016), to identify functional domains; and (b) reciprocal best hit BLAST v2.3.0 searches against the Swiss‐Prot database (version of Sep 27, 2017), to identify putative orthologs in model organisms. In both cases, an e‐value cutoff of 1e^−5^ was applied, providing 14,705 (72% of 20,461) and 5,393 (26%) genes with Pfam and Swiss‐Prot annotations, respectively. Genes of particular interest (i.e., DEGs and high‐connectivity genes) without reciprocal Swiss‐Prot hits were unidirectionally mapped to the Swiss‐Prot and TrEMBL databases (versions of Feb 16, 2018) with e‐value ≤1e^−10^, identity ≥40%, and length ≥75 bp cutoffs. Genes without Swiss‐Prot and Pfam hits were considered uncharacterized. Gene Ontology (GO) terms associated with Pfam hits were obtained from the pfam2go mapping file (version of Feb 18, 2017) supplied by InterPro (www.ebi.ac.uk/interpro). Hypergeometric *p*‐values for overrepresentation of GO terms in gene sets of interest were calculated by the R packages GOstats v2.46.0 and GO.db v3.6.0. Only Biological Process terms with *p*‐values <0.05 and represented by at least 2% of the total number of genes in the gene set were considered. Redundant terms with a semantic similarity score (simRel) ≥0.5 were removed with REVIGO (Supek, Bošnjak, Škunca, & Šmuc, 2011).

### 2.6 Genetic differentiation

Genome‐wide estimates of *F*_ST_ values between sample groups (Wai‘ōpae vs. Kīholo, clade C vs. clade D, and clade C vs. clade D from Wai‘ōpae only) were calculated from synonymous SNPs identified in the RNA‐seq reads making up the default dataset, excluding GA‐affected samples (*n* = 17). SNPs were called using the Samtools/BCFtools pipeline (Li, 2011; Li et al., 2009), implementing Bowtie2 (Langmead & Salzberg, 2012) to map lightly trimmed reads with a minimum mapping quality score (MAPQ) of 20 to the coral host transcriptome reference. Preliminary SNP calls were subjected to the following filters: minor allele frequency of 0.1, maximum allowable missing data threshold of 0.95, and a minimum depth of 20. Of the resulting 46,307 SNPs, 12,287 were accepted as synonymous using SnpEff (Cingolani et al., 2012) by comparison to open reading frames identified with TransDecoder (Haas et al., 2013). Extracting only a single SNP per transcript by selecting the first occurrence in the longest isoform reduced this number to 2,524 (dataset is available from the Dryad Digital Repository: https://doi.org/10.5061/dryad.db4r63m). Average pairwise *F*_ST_ values between samples of different groups were calculated from these remaining SNPs according to Weir and Cockerham (1984), using the genet.dist function (method = “WC84”) in hierfstat (Goudet, 2005). Confidence intervals were obtained from 1,000 bootstrap replicates with the boot.ppfst function.

## 3 RESULTS

### 3.1 *Symbiodinium *clade and host genetic differentiation

We examined the composition of the *Symbiodinium *community in each sample by extracting ITS2 sequences from the meta‐transcriptome assembly and individual dataset mapping. The two reference transcripts in the assembly were identified as belonging to clade C, type C31, and to clade D, type D1a (*Symbiodinium trenchii*). In 22 of 27 samples, raw reads only mapped to either the C31 or the D1a reference (Table 1; Supporting Information Table S1). The remaining five samples were characterized by a mix of both clades, with the more frequent clade contributing 67%–91% of the ITS2 reads. The majority of samples, especially among those assigned to clade D, contained reads that did not perfectly match the reference (see Supporting Information Table S1). In these cases, the number of mismatched reads ranged from 7% to 44% of all mapped reads per sample (averages: clade C = 11%, clade D = 24%), and typically manifested as a single‐nucleotide difference, or more rarely, as several differences at the 3′ end of the read. This suggests that most mismatched reads represented sequencing errors, intragenomic variants, or alternative genotypes, although we cannot exclude that some samples hosted alternative types of the same clade at low frequencies. Thus, the symbiont community in each colony appeared to be dominated by *Symbiodinium *from a single clade (C or D), and types C31 and D1a specifically.

**Table 1:** Sampling site, dominant *Symbiodinium *clade and Growth Anomaly (GA) disease status of *Montipora capitata *samples used in this study

Sample	Site	Clade^a^	GA status^b^
KH1	Kīholo	D	Healthy
KH2	Kīholo	D	Healthy
KH8	Kīholo	D	Healthy
KU2	Kīholo	C	GA, unaffected
KU3	Kīholo	D	GA, unaffected
KU5	Kīholo	D	GA, unaffected
KA2	Kīholo	C	GA, affected
KA3	Kīholo	D	GA, affected
KA5	Kīholo	D	GA, affected
WH1	Wai‘ōpae	C	Healthy
WH2	Wai‘ōpae	D	Healthy
WH3	Wai‘ōpae	C	Healthy
WH5	Wai‘ōpae	D	Healthy
WH8	Wai‘ōpae	C: 0.33, D: 0.67	Healthy
WH12	Wai‘ōpae	D	Healthy
WU1	Wai‘ōpae	C	GA, unaffected
WU2	Wai‘ōpae	C	GA, unaffected
WU3	Wai‘ōpae	D	GA, unaffected
WU5	Wai‘ōpae	C	GA, unaffected
WU8	Wai‘ōpae	C	GA, unaffected
WU12	Wai‘ōpae	D: 0.91, C: 0.09	GA, unaffected
WA1	Wai‘ōpae	C: 0.88, D: 0.12	GA, affected
WA2	Wai‘ōpae	C	GA, affected
WA3	Wai‘ōpae	D: 0.87, C: 0.13	GA, affected
WA5	Wai‘ōpae	C	GA, affected
WA8	Wai‘ōpae	C	GA, affected
WA12	Wai‘ōpae	D: 0.62, C: 0.38	GA, affected

Notes

GA: Growth Anomaly.

a Clade composition was determined by ITS2 transcript abundance; “C” and “D” indicate only symbionts of one clade were detected, otherwise estimated relative frequencies for each clade are given (see also Supporting Information Table S1 for absolute read counts and library sizes).

b “GA, affected” and “GA, unaffected” refers to lesioned and morphologically unaffected tissue sampled from GA‐diseased colonies.

Colonies from Wai‘ōpae were equally likely to house either clade (C = 6, D = 6), while most colonies from Kīholo were associated with clade D (C = 1, D = 5). The association between clade composition and site was not significant (Yates's chi‐square of independence, *X* ^2^ = 1.87, *p*‐value = 0.17). Regarding GA, a higher prevalence of clade D was observed in healthy colonies (C = 2, D = 7) than in diseased colonies (C = 5, D = 4), although this association was also not significant (Yates's chi‐square of independence, *X* ^2^ = 2.10, *p*‐value = 0.15). Clade composition did not differ between affected and unaffected tissue in the same colony, except in some cases where clade composition in affected tissue was observed to shift away from the more homogeneous state in unaffected tissue, resulting in a mixed composition (Table 1).

Host genetic differentiation was estimated from 2,524 synonymous SNPs obtained by mapping individual RNA‐seq datasets against the coral host transcriptome reference. Between samples from the two field sites, Wai‘ōpae and Kīholo, the genome‐wide *F*_ST_ averaged 0.046 (confidence interval 0.040–0.052). Between samples dominated by different *Symbiodinium *clades, the *F*_ST_ was 0.036 (CI 0.031–0.041) across all samples, and 0.058 (CI 0.049–0.065) for samples from Waiʻōpae only.

### 3.2 Differential gene expression

**Figure 1:**
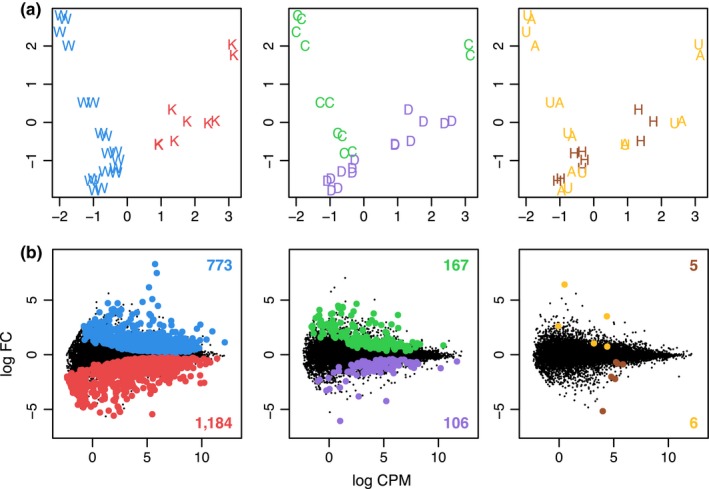
Gene expression profiles and differential gene expression of *Montipora capitata *hosts visualized by metric multidimensional scaling (MDS) and mean‐difference (MD) plots. (a) MDS plots show overall similarity in gene expression between all samples, which are indicated by letters according to field site (W = Wai‘ōpae, K = Kīholo), dominant *Symbiodinium *clade (C, D), and GA disease status (H = healthy, A = GA‐affected, U = GA‐unaffected). The distance between each pair of samples represents the typical log_2_ fold change between those samples with respect to gene expression. (b) MD plots depict average log_2 _expression in counts per million (CPM) across samples, and log_2_ fold change between groups in pairwise comparisons (default dataset). Differentially expressed genes (DEGs, *p* < 0.05) are highlighted, using the same color code for groups as in panel A. Numbers refer to DEGs expressed at higher levels in each group with respect to the other. To study colony‐level effects in GA‐diseased colonies, A and U samples were combined.

Metric MDS of gene expression profiles revealed a clear separation of samples by field site (Wai‘ōpae vs. Kīholo). In addition, samples were found to cluster by dominant *Symbiodinium *clade (Figure 1a; Supporting Information Figure S1). Gene expression profiles of samples associated with clade D appeared more uniform than those with clade C, which were more spread out. As expected based on a previous study (Frazier et al., 2017), GA disease status explained less of the variation in gene expression than site and clade, with the profiles of healthy and GA‐diseased samples overlapping significantly. Notably, samples obtained from GA‐affected and GA‐unaffected tissue of the same colony were consistently found in close proximity to each other, and clustered separately from healthy samples, implying that GA‐associated changes at the transcriptome level affect the whole colony. After removing outliers, pairwise comparisons of sample groups yielded a high number of differentially expressed genes (DEGs) at *p* < 0.05 in the default dataset. Consistent with the strong separation of gene expression profiles by site, the highest number of DEGs was found between Wai‘ōpae and Kīholo, with 773 and 1,184 more highly expressed genes, respectively (total = 1,957; Figure 1b). GO term enrichment analyses in the Biological Process category revealed only a few common, nonoverlapping gene functions to be overrepresented among these genes, including organic cyclic compound biosynthetic process (61 genes, *p* = 0.021) and DNA‐templated regulation of transcription (47 genes, *p* = 0.045; Table 2).

**Table 2:** Summary of over represented Gene Ontology (GO) terms in differentially expressed genes between field sites (default dataset) and between clades (Wai‘ōpae dataset)

Contrast	GO term	Count	Size	Odds ratio	*p*‐value
Site	Organic cyclic compound biosynthetic process	61	455	1.4	0.021
	Aromatic compound biosynthetic process	58	428	1.4	0.002
	Heterocycle biosynthetic process	58	436	1.4	0.028
	Transcription, DNA‐templated	47	353	1.3	0.045
	Regulation of metabolic process	47	311	1.6	0.005
Clade	Macromolecule metabolic process	25	1918	1.8	0.027
	Cellular macromolecule metabolic process	24	1539	2.3	0.003
	DNA metabolic process	10	408	3.1	0.004
	Phosphorus metabolic process	9	507	2.1	0.043
	DNA integration	7	241	3.5	0.007
	Protein phosphorylation	7	356	2.3	0.046
	Nitrogen compound transport	4	131	3.5	0.033
	Cation transport	4	135	3.4	0.036

Note

For each GO term, the number of genes observed in the gene set (count), the total number of genes in the coral host transcriptome representing this GO term (size), and hypergeometric test results for overrepresentation (odds ratio and *p*‐value) are given.

**Figure 2:**
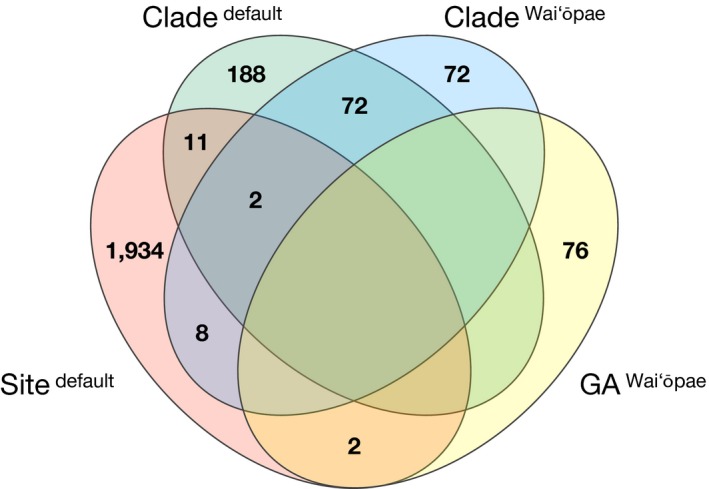
Venn diagram depicting the overlap between differentially expressed host genes in comparisons of field site (Wai‘ōpae vs. Kīholo), dominant *Symbiodinium *clade (C vs. D), and GA disease status (healthy vs. GA‐diseased). Comparisons were based on two different sets of samples: the default dataset, and a subset consisting only of samples from Wai‘ōpae. Empty fields indicate no overlap was found.

A moderate number of DEGs was also detected between samples housing different clades, with 167 and 106 genes expressed at higher levels in clade C and D samples, respectively (total = 273; Figure 1b). In contrast, testing for DEGs between healthy and GA‐diseased samples (GA‐affected and GA‐unaffected tissue combined) resulted in only 11 genes total. To examine the effects of *Symbiodinium *clade on host gene expression in more detail, we removed environmental effects by looking at a subset of data consisting only of samples from Wai‘ōpae. This approach yielded 80 genes more highly expressed in clade C, and 74 in clade D hosts. GO term analysis of these 154 genes showed enrichment of several terms pertaining to metabolic processes, including DNA metabolic process (10 genes, *p* = 0.004) and protein phosphorylation (seven genes, *p* = 0.046), as well as DNA integration (seven genes, *p* = 0.006) and transport processes (total eight genes, *p* ≤ 0.003; Table 2). About half of these 154 genes (*n* = 74) overlapped with the 273 DEGs observed between clade C and D in the default dataset above, whereas little to no overlap was found between any other comparisons (Figure 2). Regarding GA disease status, considering samples from Wai‘ōpae only increased the number of genes expressed at higher levels in healthy samples to 17, and in GA‐diseased samples to 61 (78 DEGs total).

### 3.3 Coexpression network analysis

**Figure 3:**
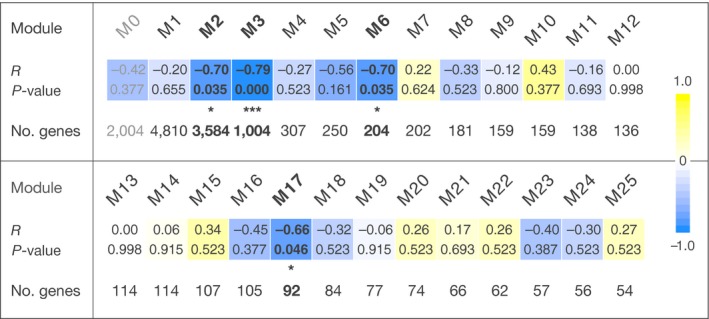
Weighted correlation network analysis (WGCNA): modules of coexpressed host genes in samples from Wai‘ōpae, and corrrelation of module gene expression profiles with differences in *Symbiodinium *clade. The strength of correlation is highlighted on a gradual color scale from yellow (*R* > 0) to blue (*R* < 0). Modules with significant correlation (FDR‐adjusted *p*‐values <0.05) are shown in bold and marked with asterisks. M0 (grayed out) includes all genes that could not be assigned to a module.

Patterns of host gene expression, and how they relate to external factors including *Symbiodinium *clade, were also examined with a system genetics approach. In the default dataset, we detected 17 modules, or groups of coregulated genes, ranging in size from 38 to 5,302 genes (Supporting Information Figure S2). In addition, a sizable number of genes (*n* = 3,079) could not be assigned to any module. According to linear regression analysis, three and four of these modules were significantly correlated with site (*R* ^2^ = 0.28–0.51) and clade (*R* ^2^ = 0.28–0.63), respectively (Supporting Information Table S2). No correlation was found between modules and GA disease status. Site‐associated modules L1 and L4 were enriched in GO terms related to biosynthetic processes (314 genes, *p* < 0.001), gene expression/RNA metabolism (>200 genes, *p* < 0.001), and others (Table 3; L15 was excluded from this analysis due to its insufficient size). Clade‐associated modules were examined in more detail in a second WGCNA analysis based on the Wai‘ōpae dataset. Unbiased by field site‐related effects, this analysis yielded a more fine‐grained network consisting of 25 modules, ranging from 54 to 4,810 genes in size (Supporting Information Figure S3). Of these, four modules proved to be significantly correlated with clade (Figure 3): M2 (3,584 genes, *R* ^2^ = 0.49, *p* = 0.035), M3 (1,004 genes, *R* ^2^ = 0.63, *p* < 0.001), M6 (204 genes, *R* ^2^ = 0.49, *p* = 0.035), and M17 (92 genes, *R* ^2^ = 0.44, *p* = 0.046). While modules M6 and M17 were too small to analyze over‐represented gene functions, module M2 was predominantly characterized by an overrepresentation of genes involved in cellular (636 genes, *p* = 0.004) and protein metabolic processes (239 genes, *p* < 0.001)—notably, protein phosphorylation (103 genes, *p* < 0.001)—as well as DNA‐templated regulation of transcription (84 genes, *p* = 0.006), and intracellular signal transduction (95 genes, *p* < 0.001), among others (Table 3). Various metabolic processes were also identified for module M3, for example, protein metabolic process (60 genes, *p* = 0.003) and phosphorus metabolic process (41 genes, *p* < 0.001), as well as DNA‐templated regulation of transcription (27 genes, *p* = 0.006) (Table 3).

**Table 3:** Summary of overrepresented Gene Ontology (GO) terms in WGCNA modules significantly correlated with field site (L1, L4; default dataset) and clade (M2, M3; Wai‘ōpae dataset)

Module	GO term	Count	Size	Odds ratio	*p*‐value
L1 (site)	Metabolic process	1,074	3,107	1.5	0.000
	Biosynthetic process	314	832	1.4	0.000
	Gene expression	247	635	1.5	0.000
	Macromolecule biosynthetic process	226	613	1.3	0.001
	RNA metabolic process	205	516	1.5	0.000
	Oxidation–reduction process	196	518	1.4	0.000
	Organic cyclic compound biosynthetic process	172	455	1.4	0.001
	Heterocycle biosynthetic process	164	436	1.4	0.002
	Aromatic compound biosynthetic process	158	428	1.3	0.005
	Organonitrogen compound biosynthetic process	109	300	1.3	0.028
	Cellular component organization or biogenesis	108	253	1.7	0.000
L4 (site)	Biological regulation	97	1,398	2.0	0.000
	Regulation of biological process	95	1,370	2.0	0.000
	Organic substance biosynthetic process	45	782	1.4	0.039
	RNA metabolic process	34	516	1.6	0.012
M2 (clade)	Cellular process	636	3,234	1.2	0.004
	Cellular macromolecule metabolic process	311	1539	1.2	0.024
	Biological regulation	286	1,398	1.2	0.017
	Organonitrogen compound metabolic process	257	1,182	1.3	0.001
	Protein metabolic process	239	1,037	1.4	0.000
	Macromolecule modification	176	607	2.0	0.000
	Phosphorus metabolic process	138	507	1.8	0.000
	Protein phosphorylation	103	356	1.9	0.000
	Intracellular signal transduction	95	292	2.2	0.000
	Heterocycle biosynthetic process	95	436	1.3	0.040
	Aromatic compound biosynthetic process	93	428	1.2	0.045
	Transcription, DNA‐templated	84	353	1.4	0.006
M3 (clade)	Organic substance metabolic process	124	2,344	1.3	0.022
	Primary metabolic process	123	2,273	1.4	0.001
	Nitrogen compound metabolic process	110	2085	1.3	0.038
	Cellular macromolecule metabolic process	86	1539	1.3	0.002
	Organonitrogen compound metabolic process	69	1,182	1.4	0.016
	Protein metabolic process	60	1,037	1.4	0.003
	Macromolecule modification	44	607	1.8	0.001
	Phosphorus metabolic process	41	507	2.0	0.000
	Protein phosphorylation	31	356	2.1	0.000
	Organic cyclic compound biosynthetic process	31	455	1.6	0.017
	Aromatic compound biosynthetic process	28	428	1.5	0.036
	Heterocycle biosynthetic process	28	436	1.5	0.044
	Transcription, DNA‐templated	27	353	1.8	0.006
	Regulation of metabolic process	26	311	2.0	0.002

Note

For each GO term, the number of genes observed in the gene set (count), the total number of genes in the coral host transcriptome representing this GO term (size), and hypergeometric test results for overrepresentation (odds ratio and *p*‐value) are given.

To further converge on individual host genes that might be particularly responsive to symbiont clade, we analyzed the connectivity of genes—that is, the extent to which their expression is correlated with that of other genes. Of the 100 most connected genes (by kTotal value), 77 were discovered to belong to clade‐associated module M2. The remainder was assigned to module M1, which was not significantly correlated with clade. None of these 100 genes overlapped with the 154 between‐clade DEGs (Supporting Information Table S3), although most DEGs were members of one of the modules significantly correlated with clade, with 61 genes belonging to M3, 40 genes to M2, five genes to M6, and two genes to M17 (29 genes could not be considered as they failed to meet the more stringent gene expression threshold of the network analysis). Most of the highly connected genes within clade‐associated modules (top 10 by kWithin value) could be annotated with Pfam domains and matched to putative Swiss‐Prot orthologs for further evaluation of gene and module function (Supporting Information Table S4).

## 4 DISCUSSION

### 4.1 Symbiodinium composition

Our primary objective was to evaluate the effects of *Symbiodinium *types from different clades on host gene expression in *M. capitata*, and identify groups of genes potentially involved in the maintenance and regulation of different symbiotic relationships. We also considered two other variables—samples were collected from sites on two opposite sides of Hawai‘i Island, and from colonies and tissues unequally affected by GA, a widespread coral disease we focused on in a previous publication (Frazier et al., 2017). At an average sequencing depth of 25 million paired‐end reads per holobiont, we were able to identify the dominant *Symbiodinium *type (C31 or D1a) in each colony from ITS2 transcript abundance, with only a minority of samples hosting both types (Table 1). At the individual and population level, this *Symbiodinium *community composition is congruent with previous studies conducted at Wai‘ōpae (Burns, Gregg, & Takabayashi, 2013), and in a *M. capitata *population on nearby O‘ahu Island (Stat et al., 2011). Reads not perfectly matching one of the references typically diverged only very slightly, suggesting they did not originate from additional *Symbiodinium *types, but rather represent sequencing errors, intragenomic variants, or alternative genotypes (compare Cunning, Gates, & Edmunds, 2017). However, determining the *Symbiodinium *community composition directly from RNA‐seq data comes with a few limitations. Short reads (75 bp in the present study) may not always span diagnostic nucleotide positions, and low coverage of symbiont transcripts can mean that rare variants (including of alternative clades) remain undetected. Further limitations stem from the nature of the ITS2 marker itself, which is not sufficiently variable to distinguish between all types, and is prone to intragenomic variation, both of which complicates the assessment of *Symbiodinium *diversity (Stat et al., 2011). However, while we may have underestimated the contribution of alternative types by our approach, it is evident the study population is dominated by symbionts from two different clades (C and D), and types C31 and D1a specifically. The impact of rare and likely transient background symbionts on host functions is contentious (Lee et al., 2016; Ziegler, Eguíluz, Duarte, & Voolstra, 2018), and differences in holobiont properties have been widely recognized in corals hosting symbionts from divergent clades. Accepting some uncertainty in the symbiont composition at the type level, we therefore focus on transcriptional effects associated with clade C and D symbionts, mainly represented by types C31 and D1a.

### 4.2 Host gene expression profiles

Across 27 RNA‐seq datasets from 18 colonies, we detected distinctly different transcriptional responses to the three variables described above. According to overall transcriptional profiles (Figure 1a) and DGE analysis (Figure 1b), the field site had the largest effect on host gene expression, followed by symbiont clade, and then GA disease status. With a clear profile separation according to MDS ordination, and 1,957 differentially expressed genes (9.6% of all 20,461 host transcripts), the effect of field site proved to be almost an order of magnitude larger than that of symbiont clade, which corresponded to 273 DEGs (1.3%) in the default dataset. Because the two sites at Waiʻōpae and Kīholo are separated by more than 200 km along the coast (125 km linear distance), we considered whether the samples represented two different populations with limited gene flow between them, resulting in gene expression differences due to contrasting genomic backgrounds. We calculated a genome‐wide *F*_ST_ of 0.046 (confidence interval 0.040–0.052) from synonymous SNPs, indicating moderate genetic differentiation between Waiʻōpae and Kīholo. *F*_ST_ values in this range have also been reported in other coral species with comparable life histories across even larger distances, using neutral and non‐neutral markers (Ayre & Hughes, 2004; Polato, Concepcion, Toonen, & Baums, 2010). Gene expression differences between sites might therefore be driven by underlying genetic variation in transcriptional regulators due to local adaptation. They may also represent a phenotypic response (acclimatization), for example, through epigenetic effects or polyphenism. Conceivably, the environment at the two field sites differed sufficiently to induce both adaptation and acclimatization, especially in a sessile organism. For instance, as a typical fringing reef on Hawai‘i Island's arid Western side, Kīholo is characterized by stable conditions with constant sea water circulation and little rainfall. In contrast, Waiʻōpae on the Eastern side received higher rainfall and groundwater influx and was defined by its complex underwater topography with partially submerged tide pools of different size and connectivity to the open ocean. Waiʻōpae was therefore subject to much higher fluctuations in temperature and salinity (Wiegner et al., 2016) than Kīholo, necessitating different short‐ and long‐term physiological responses. The magnitude of gene expression differences between sites was comparable to those in a similar study, which reported about 3,000 DEGs between two sites (Barfield et al., 2018) in Australian *A. millepora*. These genes showed enrichment in macromolecule biosynthesis and RNA processing functions, suggesting some overlap with our GO term analysis, which highlighted cyclic compound biosynthesis and transcription as potentially important functions in response to different environments (Table 2).

Finally, we found no clustering of samples by GA disease status (Figure 1a), and only 11 DEGs between healthy and diseased colonies (Figure 1b). This number was likely deflated by superseding effects of site and symbiont type, which may have led to a decrease in the signal‐to‐noise ratio. Indeed, the number of DEGs went up to 78 after removing all samples from Kīholo. This magnitude of DGE, and the pattern of colony‐level response to GA, was consistent with the results of our previous study based on the same samples from Waiʻōpae (Frazier et al., 2017).

### 4.3 Site‐controlled clade effects

We found little to no overlap in DEGs obtained from comparisons based on site, clade, and GA disease (Figure 2), and in coexpression modules correlated with these variables (Supporting Information Table S2). This suggests that gene expression responses to site, clade, and GA disease are largely independent of each other, despite the overrepresentation of clade D in Kīholo and healthy samples, respectively. Because the sampling site was found to be a significant factor and clade C was underrepresented at Kīholo (Figure 1a), we ran a more focused analysis with only Waiʻōpae samples to obtain a more accurate picture of gene expression changes associated with symbiont clade. This decreased the number of clade‐dependent DEGs to 154, slightly more than half of which were also found in the 273 DEGs observed in the default dataset (Figure 2). While moderate in comparison with transcriptome size (0.8%), this number suggests a correlation between the composition of the *Symbiodinium *community and the transcriptional state of the host. To our knowledge, differences in host gene expression associated with different *Symbiodinium* types in established symbioses have been reported by only a handful of studies before (Barfield et al., 2018; DeSalvo et al., 2010; Matthews et al., 2017). These observations are intriguing as they open possibilities to examine host–symbiont genetic interactions that maintain and regulate different symbiotic relationships. However, unraveling the nature and cause of correlation between host gene expression and symbiont type is challenging. For instance, host genotype might influence which symbiont types are able to establish themselves in the host, so that both host gene expression and symbiont type are ultimately correlated with host genotype. To test this, we estimated genome‐wide genetic differentiation between hosts harboring clade C and clade D. With *F*_ST_ = 0.036 (CI 0.031–0.041) across all samples and 0.058 (CI 0.049–0.065) for Waiʻōpae only, we found limited but potential evidence for systematic host genotypic differences that might drive the distribution of symbiont types. Studying field samples, we were not able to take advantage of host clones to control for such effects of host genotype, as were DeSalvo et al. (2010 **)** and Matthews et al. (2017). However, the *Symbiodinium *community composition has been shown to be uncorrelated with host genotype in a geographically nearby population of *M. capitata* (Stat et al., 2011, based on ATP synthase subunit beta intron—but see Quigley, Willis, & Bay, 2017) and is likely influenced by microhabitat conditions, in particular light exposure (Innis, Cunning, Ritson‐Williams, & Gates, 2018). Considering that symbiotic interactions require a complex dialog between partners, it is also conceivable—even likely—that *Symbiodinium* symbionts and their coral hosts have evolved the ability to modulate each other's transcriptional activity. For instance, hosts seem to be able to influence physiological responses in their symbionts (Parkinson, Banaszak, Altman, LaJeunesse, & Baums, 2015). Conversely, DeSalvo et al. (2010 **)** speculated that host physiology may respond to different symbiont types, or that *Symbiodinium *may be able to explicitly influence host gene expression and physiology. Gene regulation following transfer of small RNAs between pathogen and host, or between mutualistic partners, has for instance been documented in plant–microbe interactions (Lelandais‐Brière, Moreau, Hartmann, & Crespi, 2016; Weiberg et al., 2013). Finally, the gene expression patterns observed here may also be a reflection of differences in host physiology associated with symbiont type. Coral colonies predominantly harboring *Symbiodinium *types of clade C or D can differ markedly with regard to several important properties. Colonies dominated by clade D generally grow more slowly (Jones & Berkelmans, 2010; Little, Oppen, & Willis, 2004; Mieog et al., 2009), which may be linked to differences in nutrient transfer between symbiont and host (Loram et al., 2007; Matthews et al., 2017). *Symbiodinium *types in clades C and D may also be adapted to different light regimes, and influence the vertical distribution pattern of their hosts (Iglesias‐Prieto et al., 2004). Most notably, some clade D symbionts confer increased resistance to elevated temperatures to the host, and reduce its susceptibility to coral bleaching (Berkelmans & van Oppen, 2006; Mieog et al., 2009; but see e.g., Hume et al., 2015). As the abundance of clade D symbionts has been increasing globally in habitats impacted by bleaching and across a wide range of host species, some are suspected to be opportunists capable of outcompeting and replacing optimally coadapted symbionts (e.g., in clade C) in health‐compromised corals (Stat & Gates, 2011). However, our experimental design does not allow us to distinguish between the host merely responding to differences in nutrient transfer, photosynthetic function, and other *Symbiodinium*‐associated traits, or direct modulation of gene expression by the symbionts. Parallel sequencing of small RNA and conventional RNA‐seq, together with advances in host–symbiont transcript separation, or experiments inducing symbiont shuffling under controlled conditions, could allow addressing this issue in future studies.

### 4.4 Key genes and coexpression networks

The magnitude of gene expression differences (e.g., low hundreds of genes) associated with variation in symbiont composition was similar to that reported from several other cnidarian host species. For instance, Matthews et al. (2017) observed roughly 100 DEGs characterizing sea anemones with normal and opportunistic *Symbiodinium *clades using a comparable RNA‐seq approach. Studies using microarrays reported similar absolute numbers, for example, DeSalvo et al. (2010) in the coral *M. faveolata*, and Boutet et al. (2011) in associations of deep‐sea mussel and chemoautotrophic bacteria. However, because these microarrays represented only a small fraction of the host transcriptome, the effect size is not fully comparable to the present study. In contrast, Barfield et al. (2018) found more than 3,000 DEGs between corals associated with different symbionts, possibly because of this study's extra level of sensitivity due to its common garden design. Among the DEGs we identified in coral hosts harboring different *Symbiodinium *clades, genes involved in DNA metabolism and integration, protein phosphorylation, and transport processes were significantly over‐represented (Table 2). Individual genes with the most significant expression differences between clades included genes encoding two transcription elongation factors, a putative but fragmentary Toll‐like receptor (TLR), collagen and ankyrin repeat‐containing proteins (the former possibly involved in Wnt signaling), and a pore‐forming cytotoxin homologous to Delta actitoxin Aas1a. We detected few transcriptional differences in genes associated with the immune system and stress response (e.g., in lectins, additional TLRs, antioxidants)—differences which might be expected when comparing an optimally coadapted (clade C) and a more opportunistic symbiont (clade D), as observed by Matthews et al. ( 2017) in sea anemones. Similarly, genes involved in translation and protein folding/degradation seemed to play less of a role than reported by DeSalvo et al. (2010). However, while DGE remains the most straightforward approach to study transcriptional responses, not all biologically relevant genes may vary significantly in expression under different conditions. Most genes are organized into networks governing cellular processes or pathways, and the activity of such a network may change by the accumulation of gene expression differences that are not detectable individually. We therefore complemented our DGE analysis with the more systemic approach of reconstructing gene networks from coexpression data (i.e., correlation in gene expression). In the default and the Waiʻōpae dataset, we identified 17 and 25 modules of coexpressed genes, respectively, which may correspond to biological pathways or shared cellular functions. Modules whose eigengenes (first principal component) were significantly correlated with site (default dataset only) and *Symbiodinium *clade (both datasets; Supporting Information Table S2; Figure 3), suggested that these gene networks play a role in biological processes affected by environmental factors and symbiont clade. Consistent with the DEGs between sites, two of the site‐dependent modules appeared to revolve around the production of macromolecules and organic cyclic compounds, as well as RNA metabolism. Focusing on clade effects in the smaller dataset, two of the modules significantly correlated with clade (M6 and M17) did not produce meaningful GO term results likely due to their small size. However, some information could be gleaned from the 10 most highly connected genes in each module, which represent hub gene candidates that may influence the activity of a disproportionate number of genes in the network (Supporting Information Tables S3 and S4). In module M6, hub gene candidates included serine proteases, as well as genes potentially involved in DNA repair and cytoskeleton regulation (e.g., homologs of Mediator of DNA damage checkpoint protein 1, spectrins). Potential hub genes in module M17 comprised a homolog of S‐adenosylmethionine synthase, several aminoacyl tRNA synthases, and ABC transporters, suggesting a module function in translation, membrane trafficking, and possibly immune response. The two larger of the significant modules, M2 and M3, showed enrichment in GO terms mostly pertaining to metabolic processes, as well as to a lesser extent, signal transduction and regulation of transcription (Table 3). Both modules featured a functionally diverse cast of potential hub genes. For instance, M2 hub gene candidates may be involved in endocytosis (Adaptor protein complex 2), RNA processing (e.g., La protein, Polypyrimidine tract binding protein 1), and signal transduction (e.g., Cyclic nucleotide phosphodiesterase, and a putative G protein), among others. In the case of M3, potential hub genes seemed to mostly play a role in signaling pathways and cytoskeleton regulation, comprising several kinases, putative kinase receptors, and others (e.g., STK, Ras and Zap70 homologs, Ankyrin 2, to focus on the most reliably annotated ones). Notably, module M2 also featured a disproportionate number of highly connected genes, since the majority of these genes in the entire transcriptome (top 100 by kTotal) fell into this group (while kTotal is correlated with the number of genes per module, M1 contained more genes in total but only a minority of highly connected genes). By combining DGE and coexpression network analyses, we discovered that none of the top overall connected genes overlapped with the 154 DEGs between clades, suggesting that clade‐associated genes are not central regulators in the host transcriptome. On the other hand, most DEGs (70%) were found within the modules significantly correlated with clade. Coexpression network analyses therefore pointed to more extensive, subtle changes in biological processes between hosts colonized by different clades than was observable directly through DGE analysis. In summary, these processes appeared to revolve around metabolic changes, transcription and translation, and cell signaling, which are consistent with previous observations in similar systems (DeSalvo et al., 2010; Matthews et al., 2017). However, it is important to note that the usefulness of homology‐based gene annotation and GO term analysis remains limited in nonmodel organisms, and typically rely on a small number of genes. In the present study, we were only able to annotate a quarter of all transcribed host genes with Swiss‐Prot homologs, and to identify Pfam domains (on which GO annotations were based) in three‐quarters. Sequence homology may not be a reliable predictor of functional homology across greater phylogenetic distances, and genes that cannot be annotated may represent taxonomically restricted or highly divergent genes with important functional roles (Tautz & Domazet‐Lošo, 2011; Wissler, Gadau, Simola, Helmkampf, & Bornberg‐Bauer, 2013). These concerns appeared to be particularly pronounced in the site‐dependent DEGs. Despite their high number, these genes were characterized by only four main GO terms, three of which were highly similar despite efforts to reduce term redundancy (Table 2). In addition, only four out of the 10 most significant DEGs could be functionally annotated based on sequence homology, indicating the rest may be taxonomically restricted. Such genes without homology to known genes have been linked to species‐specific adaptations in response to changing environments (Colbourne et al., 2011; Voolstra et al., 2011). It is thus conceivable taxonomically restricted genes are over‐represented in DGEs from two sites characterized by different environmental conditions, although this possibility requires further analysis.

## 5 CONCLUSIONS

Unraveling the complexity of host–symbiont interactions at the molecular level remains an important goal in the life sciences. In this study, we investigated transcriptome‐wide differences in host gene expression associated with two dominant types of *Symbiodinium *clade C and D in the coral *M. capitata*, which is of particular interest considering some clade D symbionts may convey higher resistance to elevated water temperatures and are globally increasing in prevalence. In our study population, we found that association with predominantly clade C or clade D types was correlated with measurable changes in host gene expression profiles, a moderate number of DEGs, and changes in the activity of coexpressed gene networks. However, considering the differences in physiology and performance between coral colonies harboring different types, in particular of clades C and D, the clade‐dependent effects appeared unexpectedly limited in scope. This was especially evident in comparison with the much more pronounced effects of environmental factors, which we explored through the inclusion of different field sites. Notably, we did not uncover large‐scale transcriptional changes pertaining to the immune or stress response, although increased activity of these systems might be expected from hosts confronted with less optimally coadapted symbionts of clade D *Symbiodinium*. This raises the question whether the types investigated here indeed differ significantly with regards to their role along the mutualism‐parasitism spectrum, as has been hypothesized for clades C and D (Stat & Gates, 2011). The clade‐based effects we observed, however, were consistent with previous studies, and involved changes in metabolic processes, as well as more subtle variation within several other systems, including the regulation of transcription and translation, cell signaling, and membrane trafficking. Differences in metabolic pathways and transport may be linked to differences in symbiont–host nutrient transfer (Loram et al., 2007), but to what extent transcriptional changes translate into metabolic changes remains unclear until further experiments (compare Matthews et al., 2017). Our experimental setup was also not designed to determine to what extent the correlation between host gene expression and symbiont type is caused by the host genomic background, the host responding to symbiont type, or more intriguingly, cross‐kingdom transcriptional modulation of the host by *Symbiodinium*. A promising avenue of research to this end would be to investigate the role of symbiont regulatory RNAs. Such an approach could further illuminate where the relationships between clade D symbionts and their hosts are located on the spectrum from mutualism to parasitism, which has important long‐term implications for coral reef health and conservation (Stat & Gates, 2011). By providing a candidate list of clade‐associated genes in *M. capitata*, we hope to motivate the further characterization of these genes, which all too often remain only incompletely annotated—after all, identifying and comparing unknown candidate genes that are found consistently in nonmodel organisms has the potential to make unexpected contributions to our understanding of host–symbiont interactions.

## CONFLICT OF INTEREST

None declared.

## AUTHOR CONTRIBUTIONS

MT, MF, and MH designed the research. MH, MF, and RB performed the experiments and analyzed the data. MH wrote the manuscript. All authors critically revised and approved the final manuscript.

## DATA ACCESSIBILITY

Raw reads: NCBI Sequence Reads Archive (SRA), accession numbers SRR5453739–65.

Meta‐ and coral transcriptome assembly, and per‐sample gene expression data: NCBI Gene Expression Omnibus, accession numbers GSM2579605–31.
