# Supplementary material for: Symbiont type and environmental factors affect transcriptome‐wide gene expression in the coral Montipora capitata
Source: Ecol Evol. 2018 Dec 27;9(1):378–92. doi: 10.1002/ece3.4756 (PMC6341978; doi:10.1002/ece3.4756)
Supplement: Supplementary file 1 [file ECE3-9-378-s001.docx]

Supplemental Information

Symbiont type and environmental factors affect transcriptome-wide gene expression in the coral *Montipora capitata*

Martin Helmkampf, M. Renee Bellinger, Monika Frazier, Misaki Takabayashi

Content:

– Table S1: *Symbiodinium* ITS2 read counts (page 2)

– Table S2: WGCNA modules and correlations, default dataset (page 3)

– Figure S1: Gene expression profiles of sample subsets (page 4)

– Figure S2: WGCNA dendrogram, default dataset (page 5)

– Figure S3: WGCNA dendrogram, Wai‘ōpae dataset (page 6)

– R code (page 7)**Table S1.** Absolute and relative counts of reads mapping to ITS2 reference sequences of *Symbiodinium* clades C and D.

| **Sample** | **Library** | **Counts C** | **Counts D** | **Prop. C** | **Prop. D** | **XM C** | **XM D** |
| --- | --- | --- | --- | --- | --- | --- | --- |
| **KH1** | 19,760,221 | 0 | 26 | 0.00 | 1.00 | 0 | 4 |
| **KH2** | 22,794,161 | 0 | 24 | 0.00 | 1.00 | 0 | 2 |
| **KH8** | 22,560,774 | 0 | 17 | 0.00 | 1.00 | 0 | 2 |
| **KU2** | 21,099,939 | 20 | 0 | 1.00 | 0.00 | 0 | 0 |
| **KU3** | 23,128,518 | 0 | 18 | 0.00 | 1.00 | 0 | 3 |
| **KU5** | 21,655,501 | 0 | 26 | 0.00 | 1.00 | 0 | 9 |
| **KA2** | 19,726,655 | 15 | 0 | 1.00 | 0.00 | 1 | 0 |
| **KA3** | 25,727,296 | 0 | 9 | 0.00 | 1.00 | 0 | 4 |
| **KA5** | 20,063,710 | 0 | 12 | 0.00 | 1.00 | 0 | 4 |
| **WH1** | 24,697,485 | 20 | 0 | 1.00 | 0.00 | 7 | 0 |
| **WH2** | 29,791,734 | 0 | 8 | 0.00 | 1.00 | 0 | 2 |
| **WH3** | 29,900,027 | 15 | 0 | 1.00 | 0.00 | 0 | 0 |
| **WH5** | 23,421,562 | 0 | 30 | 0.00 | 1.00 | 0 | 5 |
| **WH8** | 25,326,525 | 2 | 4 | 0.33 | 0.67 | 0 | 0 |
| **WH12** | 26,894,337 | 0 | 19 | 0.00 | 1.00 | 0 | 5 |
| **WU1** | 35,887,007 | 51 | 0 | 1.00 | 0.00 | 2 | 0 |
| **WU2** | 31,405,725 | 57 | 0 | 1.00 | 0.00 | 9 | 0 |
| **WU3** | 35,894,848 | 0 | 21 | 0.00 | 1.00 | 0 | 6 |
| **WU5** | 17,406,795 | 12 | 0 | 1.00 | 0.00 | 4 | 0 |
| **WU8** | 22,889,791 | 28 | 0 | 1.00 | 0.00 | 4 | 0 |
| **WU12** | 22,384,755 | 2 | 21 | 0.09 | 0.91 | 0 | 6 |
| **WA1** | 28,825,180 | 23 | 3 | 0.88 | 0.12 | 5 | 1 |
| **WA2** | 30,338,749 | 42 | 0 | 1.00 | 0.00 | 6 | 0 |
| **WA3** | 32,461,048 | 2 | 13 | 0.13 | 0.87 | 0 | 5 |
| **WA5** | 20,622,848 | 17 | 0 | 1.00 | 0.00 | 4 | 0 |
| **WA8** | 18,512,225 | 35 | 0 | 1.00 | 0.00 | 4 | 0 |
| **WA12** | 19,405,625 | 8 | 13 | 0.38 | 0.62 | 0 | 2 |

Note – “XM” refers to mismatched reads, which may represent intragenomic variants, sequencing errors, or the presence of alternative types or genotypes. “Library” indicates the total number of reads in each meta-transcriptome (including host and symbiont transcripts), after removing low-expression transcripts.Table S2. Modules of co-expressed coral host genes detected by weighted correlation network analysis (WGCNA) in the default dataset, and correlations with field site, *Symbiodinium* clade, and GA disease status.

| **Module** | **No. Genes** | **Site** | | | **Clade** | | | **GA** | | |
| --- | --- | --- | --- | --- | --- | --- | --- | --- | --- | --- |
|  |  | Slope | R^2^ | P-value | Slope | R^2^ | P-value | Slope | R^2^ | P-value |
| L0 | 3079 | 0.22 | 0.28 | **0.036** | -0.31 | 0.56 | **0.000** | -0.11 | 0.06 | 0.402 |
| L1 | 5302 | 0.30 | 0.51 | **0.000** | -0.07 | 0.03 | 0.583 | -0.03 | 0.00 | 0.816 |
| L2 | 2067 | 0.15 | 0.13 | 0.166 | -0.19 | 0.21 | 0.069 | -0.05 | 0.02 | 0.682 |
| L3 | 1082 | 0.16 | 0.15 | 0.166 | -0.06 | 0.02 | 0.629 | -0.09 | 0.04 | 0.453 |
| L4 | 890 | -0.22 | 0.28 | **0.036** | 0.03 | 0.00 | 0.817 | -0.01 | 0.00 | 0.952 |
| L5 | 738 | -0.09 | 0.05 | 0.367 | -0.25 | 0.36 | **0.012** | -0.14 | 0.10 | 0.396 |
| L6 | 477 | -0.11 | 0.07 | 0.292 | -0.22 | 0.28 | **0.029** | -0.11 | 0.06 | 0.402 |
| L7 | 385 | 0.14 | 0.11 | 0.184 | -0.33 | 0.63 | **0.000** | -0.11 | 0.06 | 0.402 |
| L8 | 164 | 0.16 | 0.15 | 0.166 | -0.03 | 0.01 | 0.817 | 0.04 | 0.01 | 0.772 |
| L9 | 87 | 0.13 | 0.10 | 0.184 | -0.15 | 0.13 | 0.160 | -0.08 | 0.04 | 0.469 |
| L10 | 86 | 0.15 | 0.13 | 0.166 | -0.03 | 0.01 | 0.817 | 0.23 | 0.27 | 0.090 |
| L11 | 70 | 0.13 | 0.10 | 0.184 | 0.17 | 0.17 | 0.104 | 0.20 | 0.22 | 0.126 |
| L12 | 64 | -0.16 | 0.14 | 0.166 | -0.09 | 0.05 | 0.422 | -0.14 | 0.11 | 0.396 |
| L13 | 62 | 0.05 | 0.01 | 0.631 | 0.14 | 0.11 | 0.185 | 0.10 | 0.06 | 0.402 |
| L14 | 62 | 0.13 | 0.10 | 0.184 | -0.00 | 0.00 | 0.963 | 0.22 | 0.27 | 0.090 |
| L15 | 40 | 0.29 | 0.48 | **0.000** | -0.18 | 0.20 | 0.072 | -0.12 | 0.08 | 0.402 |
| L16 | 39 | 0.05 | 0.01 | 0.631 | 0.14 | 0.12 | 0.162 | 0.13 | 0.09 | 0.401 |
| L17 | 38 | 0.03 | 0.00 | 0.776 | 0.23 | 0.30 | **0.023** | 0.14 | 0.11 | 0.396 |

Note – slope, correlation coefficient R^2^, and FDR-adjusted p-value refer to linear regression results testing for correlation between module eigengenes (gene expression profiles) and sampling site, dominant *Symbiodinium* clade, and GA disease status. Significant correlations are shown in bold. L0 (in gray) includes all genes that could not be assigned to a module. Based on the default dataset.
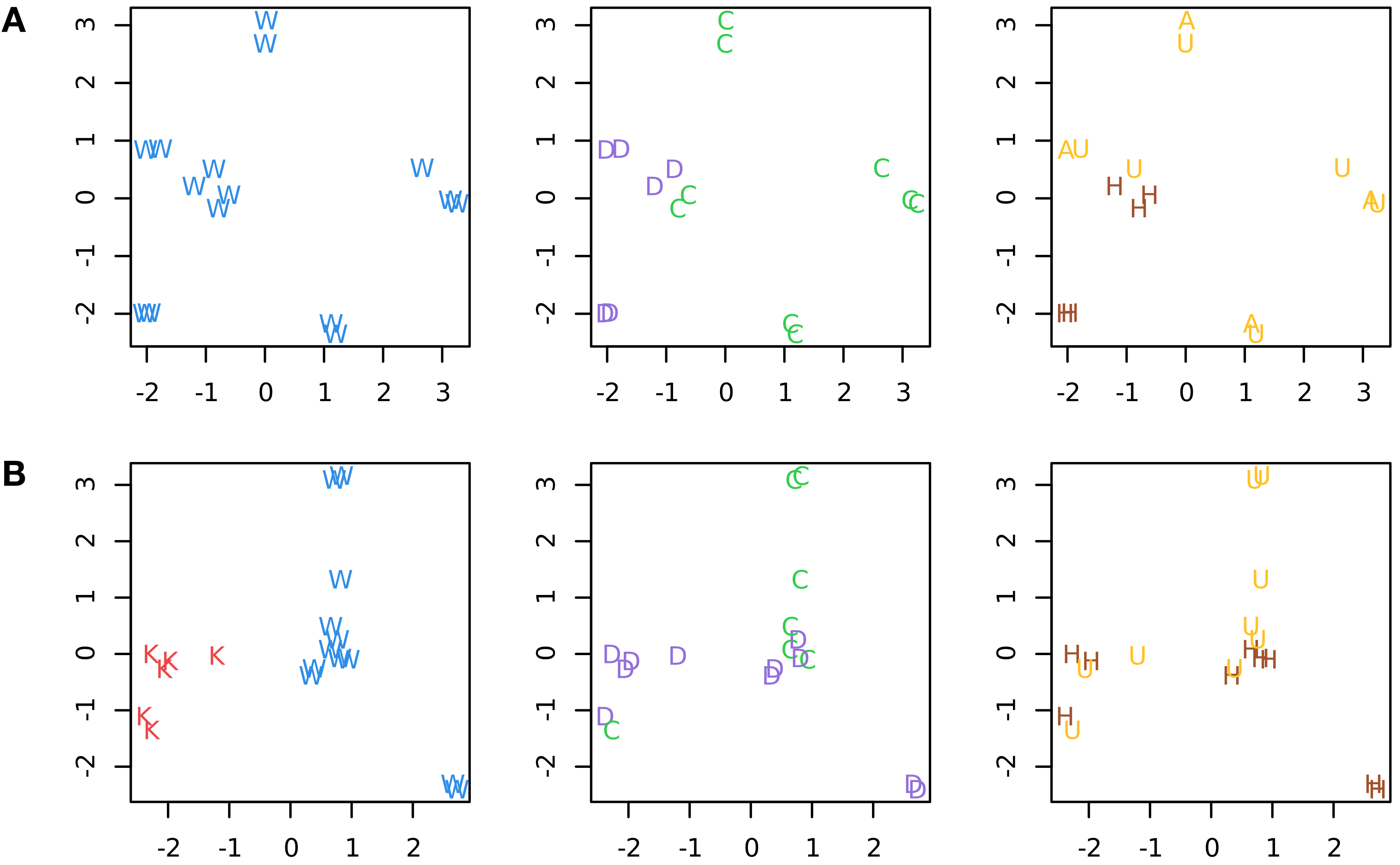


Figure S1. Metric multidimensional scaling plots showing overall similarity in gene expression between samples in the (A) Wai’ōpae and (B) HU datasets (i.e. samples from both sites, but excluding samples from GA lesions). Samples are indicated by letters according to field site (W = Wai’ōpae, K = Kīholo) dominant *Symbiodinium* clade (C, D), and GA disease status (H = healthy, A = GA-affected, U = GA-unaffected). The distance between each pair of samples represents the typical log_2_ fold change between those samples with respect to gene expression.
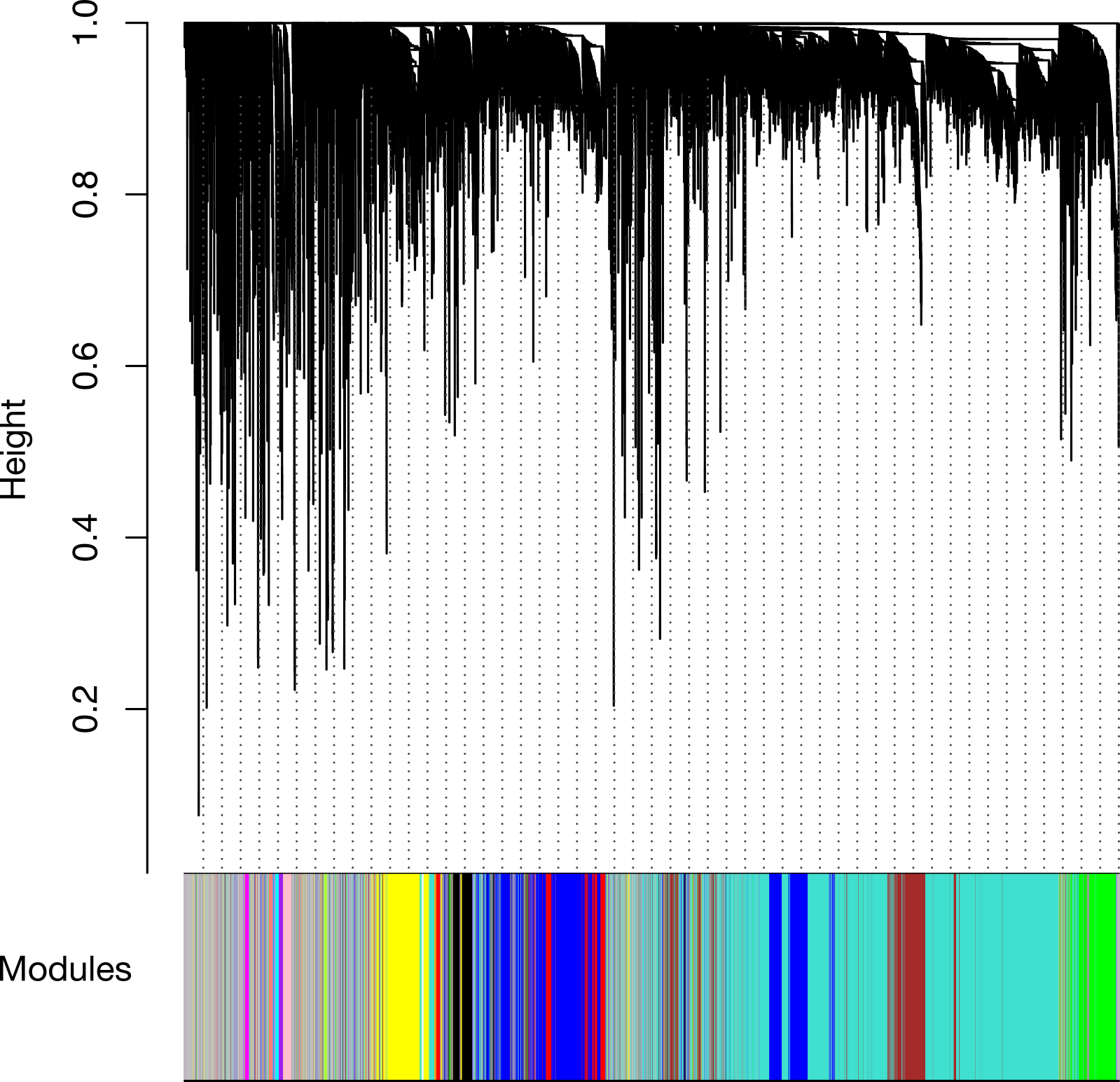


Figure S2. Hierarchical clustering dendrogram of *M. capitata* genes, constructed with WGCNA based on the default dataset. Co-expressed genes are assigned the same color, forming modules (n = 17). Genes not falling into any module are shown in gray.
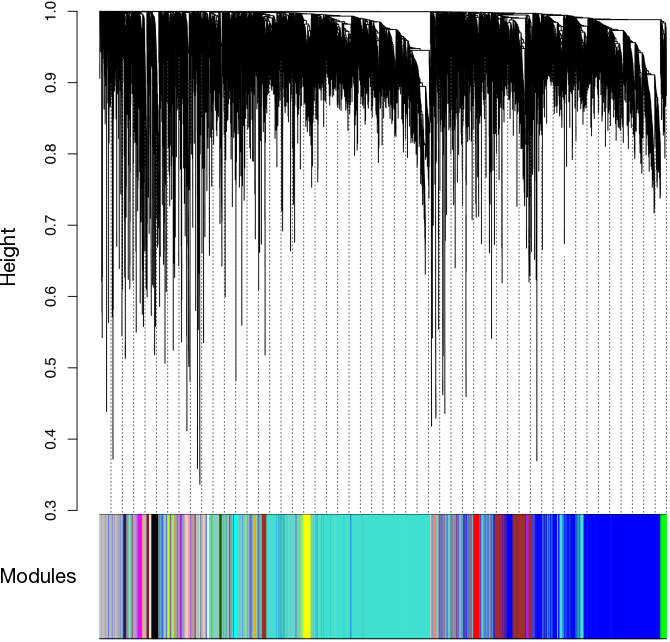


Figure S3. Hierarchical clustering dendrogram of *M. capitata* genes, constructed with WGCNA based on the Wai’ōpae dataset. Co-expressed genes are assigned the same color, forming modules (n = 25). Genes not falling into any module are shown in gray.R code

### Differential gene expression analysis ###

library(edgeR)

library(DESeq)

# Set working directory

setwd("/path")

# Import and reorder count data

mcap_all = read.csv(file = "Mcap_v3_counts.csv", header = TRUE, row.names = 1, sep = ",")

factors_all = read.csv(file = "factorsCD.csv", header = TRUE, row.names = 1, sep = ",")

mcap_all = mcap_all[ , order(names(mcap_all))]

factors_all = factors_all[order(rownames(factors_all)) , ]

# Optional: subset data

# remove outliers (WA8) and CD samples (WH8, WA12) – dataset cdo / default

mcap_cdo = mcap_all[ , c(-11,-15,-21)]

factors_cdo = factors_all[c(-11,-15,-21) , ]

# remove outliers and Kiholo – dataset W / Waiopae only

mcap_W = mcap_all[ , c(-1,-2,-3,-4,-5,-6,-7,-8,-9,-11,-15,-21)]

factors_W = factors_all[c(-1,-2,-3,-4,-5,-6,-7,-8,-9,-11,-15,-21) , ]

# remove GA-affected – dataset HU

# mcap_HU = mcap_all[ , c(-1,-2,-3,-10,-11,-12,-13,-14,-15)]

# factors_HU = factors_all[c(-1,-2,-3,-10,-11,-12,-13,-14,-15) , ]

# Assign dataset

counts = mcap_W

factors = factors_W

## Data pre-processing and quality control

# Transform counts to (log-) counts per million

cpm = cpm(counts)

logcounts = cpm(counts, log = TRUE)

# Filter noise (minimum 1 count per million reads in at least 3 samples)

thres = cpm > 1

table(rowSums(thres))

keep = rowSums(thres) >= 3

counts_keep = counts[keep,]

dim(counts_keep)

# Create DGEList object with sample information

y = DGEList(counts = counts_keep)

site = y$samples$site = factors$site

clade = y$samples$clade = factors$binary

disease = y$samples$disease = factors$disease

# Library size distribution

barplot(y$samples$lib.size, names = colnames(y), las = 2)

summary(y$samples$lib.size)

# CPM distribution

boxplot(logcounts, xlab = "", ylab = "Log2 CPM", las = 2)

abline(h=median(logcounts),col="blue")

title("Boxplots of logCPMs (unnormalized)")

# Normalize gene expression distribution (eliminate composition bias)

y = calcNormFactors(y, method = "TMM")

# replot CPM distribution after applying voom to check normalization

## Cluster samples

# Perform simple hierarchical clustering

normCounts = t(cpm(y))

distance = dist(normCounts)

clusters = hclust(distance)

dev.off()

plot(clusters)

# Multidimensional scaling (MDS) ordination (pairwise gene selection)

par(mfrow=c(2,3), mar=c(2,2,2,2), pty="s")

col.site = site

col.clade = clade

col.disease = tissue

levels(col.site) = c("#EA4749","#308EE7") # soft red, bright blue

levels(col.clade) = c("#33CC4D","#9370DB") # strong lime green, soft violet

levels(col.disease) =c("#FFC125","#A0522D","#FFC125") # goldenrod1, sienna

col.site = as.character(col.site)

col.clade = as.character(col.clade)

col.disease = as.character(col.disease)

# Print as 6" x 6" (square plot area forced)

plotMDS(logcounts, labels=site, col=col.site, dim=c(1,2))

plotMDS(logcounts, labels=clade, col=col.clade, dim=c(1,2))

plotMDS(logcounts, labels=tissue, col=col.disease, dim=c(1,2))

dev.off()

## Differential gene expression

# Set up design matrix

design_WK = model.matrix(~ 0 + site)

design_CD = model.matrix(~ 0 + clade)

design_HG = model.matrix(~ 0 + disease)

# Remove count heteroscedasticity (voom transformation)

v_WK = voom(y, design_WK, plot = FALSE)

v_CD = voom(y, design_CD, plot = FALSE)

v_HG = voom(y, design_HG, plot = FALSE)

# Optional: plot count variance before and after transformation

# par(mfrow = c(1,2))

# boxplot(logcounts, xlab = "", ylab = "Log2 counts per million", las = 2, main = "Unnormalized logCPM")

# abline(h = median(logcounts), col = "red")

# boxplot(v$E, xlab = "", ylab = "Log2 counts per million", las = 2, main = "Voom transformed logCPM")

# abline(h = median(v$E), col = "red")

# Specify contrasts

contrasts_WK = makeContrasts(

W_vs_K = siteW - siteK,

levels = colnames(design_WK)

)

contrasts_CD = makeContrasts(

C_vs_D = cladeC - cladeD,

levels = colnames(design_CD)

)

contrasts_HG = makeContrasts(

H_vs_G = diseaseH - diseaseG,

levels = colnames(design_HG)

)

# Fit linear model

fit_WK = lmFit(v_WK)

fit_WK = contrasts.fit(fit_WK, contrasts = contrasts_WK)

efit_WK = eBayes(fit_WK)

estat_WK = decideTests(efit_WK)

summary(estat_WK)

fit_CD = lmFit(v_CD)

fit_CD = contrasts.fit(fit_CD, contrasts = contrasts_CD)

efit_CD = eBayes(fit_CD)

estat_CD = decideTests(efit_CD)

summary(estat_CD)

fit_HG = lmFit(v_HG)

fit_HG = contrasts.fit(fit_HG, contrasts = contrasts_HG)

efit_HG = eBayes(fit_HG)

estat_HG = decideTests(efit_HG)

summary(estat_HG)

# Optional: apply fold-change cutoff

# tfit = treat(fit, lfc = 1) # log-FC = 1 (fold change = 2)

# tstat = decideTests(tfit)

# summary(tstat)

# List and output DE genes

deg_WK = topTable(efit_WK, p.value = 0.05, number = Inf, sort.by = "logFC")

deg_CD = topTable(efit_CD, p.value = 0.05, number = Inf, sort.by = "logFC")

deg_HG = topTable(efit_HG, p.value = 0.05, number = Inf, sort.by = "logFC")

write.csv(deg_WK, file = "DEG_WK-cdo.csv", row.names = TRUE)

write.csv(deg_CD, file = "DEG_CD-cdo.csv", row.names = TRUE)

write.csv(deg_HG, file = "DEG_HG-cdo.csv", row.names = TRUE)

write(row.names(deg_WK[deg_WK$logFC > 0 , ]), file = "DEG_Wup-cdo.ids")

write(row.names(deg_WK[deg_WK$logFC < 0 , ]), file = "DEG_Kup-cdo.ids")

write(row.names(deg_CD[deg_CD$logFC > 0 , ]), file = "DEG_Cup-cdo.ids")

write(row.names(deg_CD[deg_CD$logFC < 0 , ]), file = "DEG_Dup-cdo.ids")

write(row.names(deg_HG[deg_HG$logFC > 0 , ]), file = "DEG_Hup-cdo.ids")

write(row.names(deg_HG[deg_HG$logFC < 0 , ]), file = "DEG_Gup-cdo.ids")

# Mean-difference (average log CPM vs log FC) - print as 6" x 6" (square plot area forced)

par(mfrow=c(1,3), mar=c(2,2,2,2), pty="s")

plotMD(efit_WK, column = 1, status = estat_WK[,1], main = NULL, xlim=c(-3,13), ylim=c(-7,9), hl.col = c("#EA4749","#308EE7"), legend = FALSE, xlab = '', ylab = '')

plotMD(efit_CD, column = 1, status = estat_CD[,1], main = NULL, xlim=c(-3,13), ylim=c(-7,9), hl.col = c("#33CC4D","#9370DB"), legend = FALSE, xlab = '', ylab = '')

plotMD(efit_HG, column = 1, status = estat_HG[,1], main = NULL, xlim=c(-3,13), ylim=c(-7,9), hl.col = c("#A0522D","#FFC125"), legend = FALSE, xlab = '', ylab = '')

### Weighted Gene Co-Expression Network Analysis ###

library(WGCNA)

library(flashClust)

options(stringsAsFactors = FALSE)

enableWGCNAThreads()

# Set working directory

setwd("/path")

# Import and reorder expression and sample data

fpkm_all = read.csv(file = "Mcap_v3_fpkm.csv", header = TRUE, row.names = 1, sep = ",")

factors_all = read.csv(file = "factorsCD.csv", header = TRUE, row.names = 1, sep = ",", stringsAsFactors = TRUE)

fpkm_all = fpkm_all[ , order(names(fpkm_all))]

factors_all = factors_all[order(rownames(factors_all)) , ]

# Subset data

fpkm_cdo = fpkm_all[ , c(-11,-15,-21)] # remove CD and outliers (WA8 = 15)

factors_cdo = factors_all[c(-11,-15,-21) , ]

fpkm_W = fpkm_all[ , c(-1,-2,-3,-4,-5,-6,-7,-8,-9,-11,-15,-21)] # remove outliers and Kiholo

factors_W = factors_all[c(-1,-2,-3,-4,-5,-6,-7,-8,-9,-11,-15,-21) , ]

# exprDat = exprDat[c(1,2,5:9,12,13,15,18:21,26,27),] # remove Kiholo, CD

# factors = subset(factors, location == "W" & clade != "CD")

# Assign dataset

fpkm = fpkm_W

factors = factors_W

# Filter noise (minimum FPKM of 1 in at least 3 samples)

thres = fpkm > 1

table(rowSums(thres))

keep = rowSums(thres) >= 3

fpkm_keep = fpkm[keep,]

dim(fpkm_keep)

# Perform log transformation

logFpkm_temp = log2(fpkm_keep + 1)

logFpkm = round(logFpkm_temp, digits = 3)

# Define dataset

exprDat = as.data.frame(t(logFpkm))

# Optional: Check for outliers

clust = flashClust(dist(exprDat), method = "average")

sizeGrWindow(7,7)

par(cex = 0.6)

par(mar = c(0,6,4,0))

plot(clust, main = "Sample clustering to detect outliers", sub="", xlab="", cex.lab=1.5, cex.axis=1.5, cex.main=2)

## Estimating soft-thresholding power (note: not working in RStudio)

# Choose a set of soft-thresholding powers

powers = c(c(1:10), seq(from = 12, to=20, by=2))

# Call the network topology analysis function

sft = pickSoftThreshold(exprDat, powerVector = powers, verbose = 5)

# Plot the results:

sizeGrWindow(9,5)

par(mfrow = c(1,2))

cex1 = 0.9

# Scale-free topology fit index as a function of the soft-thresholding power

plot(sft$fitIndices[,1], -sign(sft$fitIndices[,3])*sft$fitIndices[,2],

xlab="Soft Threshold (power)",ylab="Scale Free Topology Model Fit,signed R^2",type="n",

main = paste("Scale independence"))

text(sft$fitIndices[,1], -sign(sft$fitIndices[,3])*sft$fitIndices[,2],

labels=powers,cex=cex1,col="red");

# This line corresponds to using an R^2 cut-off of h

abline(h=0.90,col="red")

# Mean connectivity as a function of the soft-thresholding power

plot(sft$fitIndices[ ,1], sft$fitIndices[,5],

xlab="Soft Threshold (power)", ylab="Mean Connectivity", type="n",

main = paste("Mean connectivity"))

text(sft$fitIndices[,1], sft$fitIndices[,5], labels=powers, cex=cex1, col="red")

## Constructing weighted co-expression network and modules (note: set exponent)

exponent = 20

net = blockwiseModules(exprDat, power = exponent,

TOMType = "unsigned", minModuleSize = 30,

reassignThreshold = 0, mergeCutHeight = 0.25,

numericLabels = TRUE, pamRespectsDendro = FALSE,

saveTOMs = TRUE,

saveTOMFileBase = "TOM",

verbose = 3,

maxBlockSize = 20000)

# Plot dendrogram (print 8" x 8")

sizeGrWindow(7,7)

mergedColors = labels2colors(net$colors)

plotDendroAndColors(net$dendrograms[[1]], mergedColors[net$blockGenes[[1]]],

"Module colors",

dendroLabels = FALSE, hang = 0.03,

addGuide = TRUE, guideHang = 0.05)

moduleLabels = net$colors

moduleColors = labels2colors(net$colors)

MEs = round(net$MEs, digits = 3)

geneTree = net$dendrograms[[1]]

table(net$colors)

# Save data

save(MEs, moduleLabels, moduleColors, geneTree,

file = "CoralWGCNA_v1.RData")

## Quantifying module-trait associations (i.e. site, clade, tissue)

# Linear regression apply

modelsS = apply(MEs, 2, function(MEs.col) lm(MEs.col ~ factors$site))

modelsC = apply(MEs, 2, function(MEs.col) lm(MEs.col ~ factors$binary))

modelsG = apply(MEs, 2, function(MEs.col) lm(MEs.col ~ factors$disease))

# Define function to extract results from list of models

lmSum = function(lm) {

out = c(lm$coefficients[1],

lm$coefficients[2],

summary(lm)$r.squared,

pf(summary(lm)$fstatistic[1], summary(lm)$fstatistic[2], summary(lm)$fstatistic[3], lower.tail = FALSE),

p.adjust(pf(summary(lm)$fstatistic[1], summary(lm)$fstatistic[2], summary(lm)$fstatistic[3], lower.tail = FALSE), method = "fdr")

)

names(out) = c("intercept","slope","r.squared","p.value","fdr")

return(out)

}

lm_S = list()

for (i in 1:length(modelsS)) lm_S[[names(modelsS)[i]]] = lmSum(modelsS[[i]])

t(as.data.frame(lm_S))

write.table(t(as.data.frame(lm_S)), file = "lm_S.csv", quote = FALSE, sep = "\t")

lm_C = list()

for (i in 1:length(modelsC)) lm_C[[names(modelsC)[i]]] = lmSum(modelsC[[i]])

t(as.data.frame(lm_C))

write.table(t(as.data.frame(lm_C)), file = "lm_C.csv", quote = FALSE, sep = "\t")

lm_G = list()

for (i in 1:length(modelsG)) lm_G[[names(modelsG)[i]]] = lmSum(modelsG[[i]])

t(as.data.frame(lm_G))

write.table(t(as.data.frame(lm_G)), file = "lm_G.csv", quote = FALSE, sep = "\t")

# write.csv(assocTable, file = "Mcap_v3-CD_assocTable.csv", quote = FALSE)

# Print modules

module2 = names(exprDat)[moduleLabels=="2"]

module3 = names(exprDat)[moduleLabels=="3"]

module6 = names(exprDat)[moduleLabels=="6"]

module17 = names(exprDat)[moduleLabels=="17"]

write(module2, file = "module2_W.ids")

write(module3, file = "module3_W.ids")

write(module6, file = "module6_W.ids")

write(module17, file = "module17_W.ids")

## Module membership (signed eigengene-based connectivity)

KME = signedKME(exprDat, MEs, outputColumnName="MM.")

head(KME)

MMcor = as.data.frame(cor(exprDat, MEs, use = "p"))

MMPvalue = as.data.frame(corPvalueStudent(as.matrix(MMcor), nrow(exprDat)))

## Intramodular connectivity

ADJ = abs(cor(exprDat, use = "p")) ^ exponent

Connectivity = intramodularConnectivity(ADJ, moduleColors)

head(Connectivity)

## Writing module membership and connectivity data to file

ModCon = cbind(round(Connectivity, digits = 3), round(KME, digits = 3), moduleLabels, moduleColors)

ModCon[ , order(names(ModCon))]

# Identify genes in interesting modules with highest intramodular connectivity (hub genes)

M2 = ModCon[moduleLabels == "2" , ]

M2.topK = M2[order(M2$kWithin, decreasing = TRUE) , ]

write(row.names(M2.topK[1:10,]), file = "M2_W_topK.ids")

M3 = ModCon[moduleLabels == "3" , ]

M3.topK = M3[order(M3$kWithin, decreasing = TRUE) , ]

write(row.names(M3.topK[1:10,]), file = "M3_W_topK.ids")

M6 = ModCon[moduleLabels == "6" , ]

M6.topK = M6[order(M6$kWithin, decreasing = TRUE) , ]

write(row.names(M6.topK[1:10,]), file = "M6_W_topK.ids")

M17 = ModCon[moduleLabels == "17" , ]

M17.topK = M17[order(M17$kWithin, decreasing = TRUE) , ]

write(row.names(M17.topK[1:10,]), file = "M17_W_topK.ids") # etc.

## Create master table combining connectivity and DGE data (run DGE script first)

deg_CD_all = topTable(efit_CD, number = Inf)

deg_CD_all = round(deg_CD[,c(1,2,5)], digits = 3)

deg_CD_all = deg_CD_all[order(rownames(deg_CD_all)) , ]

ModCon_CD = ModCon[,c("kTotal","kWithin","kOut","MM.2","MM.3","MM.6","MM.17","moduleLabels","moduleColors")]

ModCon_CD = ModCon_CD[order(rownames(ModCon_CD)) , ]

Master_CD = merge(ModCon_CD, deg_CD_all, by = "row.names", all = TRUE)

write.csv(Master_CD, file = "Master_CD_W.csv")

### GO term enrichment analysis ###

library(GO.db)

library(GOstats)

library(GSEABase)

# Set working directory

setwd("/path")

# Import and prepare GO to gene mapping

goDat = read.csv(file = "Mcap_v3i_GOpfam_map.csv", header = TRUE, stringsAsFactors = FALSE, sep = ",")

goFrame = GOFrame(goDat, organism = "Montipora capitata")

goAllFrame = GOAllFrame(goFrame)

gsc = GeneSetCollection(goAllFrame, setType = GOCollection())

# Setting up parameter object

universe = unique(goDat$gene)

# Define dataset

geneSet = "CD_W" # Adjust manually

genes = scan(file = paste(geneSet, ".ids", sep = ""), what = character(0))

# Run hypergeometric test

params = GSEAGOHyperGParams(name = geneSet,

geneSetCollection = gsc,

universeGeneIds = universe,

geneIds = genes,

ontology = "BP",

pvalueCutoff = 0.05,

conditional = FALSE,

testDirection = "over")

goTest = hyperGTest(params)

goRes = summary(goTest)

head(goRes)

write.table(goRes, paste(geneSet, "_gotest.csv", sep = ""), quote = FALSE, sep = ",")

# Apply cut-offs

ngenes = 1957 # Enter total number of genes in gene set of interest manually

goLim = subset(goRes, subset = goRes$Pvalue < 0.05)

goLim = subset(goLim, goLim$Count >= ngenes * 0.02)

goLim = goLim[order(-goLim$Count),]

head(goLim)

write.table(goLim, paste(geneSet, "_golim.csv", sep = ""), quote = FALSE, sep = ",")

write(goLim$GOBPID, paste(geneSet, "_golim.ids", sep = ""))

## Remove GO terms with semantic similarity ≥ 0.5 in REVIGO

# Filter test results

# geneSet = "CD_W" # Adjust manually

# results = read.csv(file = paste(geneSet, "_golim.csv", sep = ""), row.names = NULL, sep = ",")

revigo = scan(file = paste(geneSet, "_revigo.ids", sep = ""), what = character(0))

resRev = results[results$GOBPID %in% revigo , ]

final = resRev[, c("Term", "Count", "Size", "OddsRatio", "Pvalue")]

final$OddsRatio = round(final$OddsRatio, digits = 1)

final$Pvalue = round(final$Pvalue, digits = 3)

write.table(final, paste(geneSet, "_gofinal.csv", sep = ""), quote = FALSE, sep = "\t", row.names = FALSE)

### Genetic differentitation

library(vcfR)

library(adegenet)

library(ape)

library(hierfstat)

rm(list = ls())

setwd("/path")

## Create genind object from vcf file

# comment/uncomment to read in selected vcf file using package vcfR

# Waiopae and Kiholo combined

vcf_file = read.vcfR("Mcap_synonymous_v1.vcf.gz")

# Waiopae only

# vcf_file = read.vcfR("Waiopae_synonymous_v1.vcf.gz")

# convert vcf_file to genind and then add population information

xind = vcfR2genind(vcf_file)

# comment/uncomment to select dataset

# C versus D, Waiopae and Kiholo combined

xind$other$epid = as.character(c("D", "D", "D", "C", "D", "D", "D", "C", "D", "C", "D", "D", "C", "C", "D", "C","C"))

# Kiholo vs. Wai opae

xind$other$epid = as.character(c("K", "K", "K", "K", "K", "K", "W", "W", "W", "W", "W", "W", "W", "W", "W", "W","W"))

# C versus D, Waiopae only

# xind$other$epid = as.character(c("D", "C", "D", "C", "D", "D", "C", "C", "D", "C", "C"))

# set population

pop(xind) = factor(xind$other$epid)

pop(xind) # shows levels

## Calculate WC84 Fst and bootstrap confidence intervals

# Weir & Cockerham 1984

genet.dist(xind[,-2], method = "WC84")

# Provides a bootstrap confidence interval (over loci) for sums of the different variance

# components (equivalent to gene diversity estimates at the different levels), and

# the derived F-statistics, as suggested by Weir and Cockerham (1984).

if(require(hierfstat)){

boot.ppfst(xind, nboot = 1000, diploid = TRUE)

}
